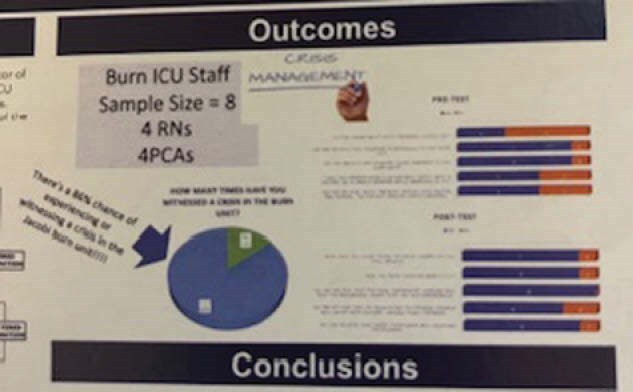# 949 De-Escalation Is Key

**DOI:** 10.1093/jbcr/iraf019.480

**Published:** 2025-04-01

**Authors:** Lazon Coleman

**Affiliations:** Jacobi Burn Center

## Abstract

**Introduction:**

De-escalation techniques are aimed at communicating effectively with an agitated patient in order to understand, manage and resolve their concerns. These actions should help reduce the patient’s agitation and the potential for aggression or violence. When an intervention is done too late or does not work it may leave the staff needing to use coercive measures to manage an aggressive or violent patient, increasing chances of injury to staff or the patient. These methods include chemical, mechanical restraints, and seclusion that can be damaging to the therapeutic relationships between patient and the provider which can also lead to harm towards patients and staff. Training de-escalation techniques to the staff has improved the interactions during crisis between the patients and staff, increasing safety for both staff and patients.

Preventing and managing crisis situations (PMCS) was used for staff development training. The patient population is filled with a variety of patients with traumatic or psychiatric history that go through crisis on the Burn Unit. A crisis is any behavior presented by an individual that can lead to harm toward self or others.

**Methods:**

The patient education department was responsible for arranging staff involvement in the PMCS training. The staff was scheduled to attend class. The staff members that attended was burn ICU RNs, the Burn Unit Head Nurse and the PCAs. I created the pre and post survey that was provided to the staff before and after attending the PMCS class. The staff participated by taking the pre and post surveys and by implementing learned skills in the unit while providing feed-back to me on how helpful the training was in maintaining safety and preventing crisis using de-escalation techniques.

**Results:**

Using a both a bar and a pie graph I showed that there was an 86% chance of witnessing a crisis on the burn unit and effective training to the staff. A staff of 8, including 4 RNs and 4 PCAs received management crisis training for the project and improved on the post exams on how to handle crisis and maintain safety for the staff and the patients.

**Conclusions:**

Showing interest, gaining awareness, and providing feedback greatly showed a need and now we have psychologist coming to the unit weekly providing assessments and visiting patients more often outside of consult request. The staff expressed that the PMCS will hopefully be standard of training for all staff across the board.

**Applicability of Research to Practice:**

This was supported by the leadership of the burn unit.

**Funding for the Study:**

No funding received or required.